# A fresh look at zebrafish from the perspective of cancer research

**DOI:** 10.1186/s13046-015-0196-8

**Published:** 2015-08-12

**Authors:** Shuai Zhao, Jian Huang, Jun Ye

**Affiliations:** Department of Surgical oncology, The Second Affiliated Hospital, Zhejiang University School of Medicine, Hangzhou, Zhejiang Province China; Department of Gastroenterology, The Second Affiliated Hospital, Zhejiang University School of Medicine, Hangzhou, Zhejiang Province China

**Keywords:** Zebrafish, Cancer model, Angiogenesis, Metastasis, Drug screen

## Abstract

Zebrafish represent a vertebrate model organism that has been widely, and increasingly, employed over the last decade in the study of developmental processes, wound healing, microbe-host interactions, and drug screening. With the increase in the laboratory use of zebrafish, several advantages, such as a high genetic homology to humans and transparent embryos, which allow clear disease evaluation, have greatly widened its use as a model for studying tumor development in vivo. The use of zebrafish has been applied in several areas of cancer research, mainly in the following domains: (1) establishing cancer models by carcinogenic chemical, genetic technology, and xenotransplantation; (2) evaluating tumor angiogenesis; (3) studying tumor metastasis; and (4) anti-tumor drug screening and drug toxicity evaluation. In this study, we provide a comprehensive overview of the role of zebrafish in order to underline the advantages of using them as a model organism in cancer research. Several related successful events are also reviewed.

## Introduction

*Danio rerio*, better known as zebrafish, have emerged as a popular model for studying developmental processes and human disorders. Zebrafish share a high level of genetic and physiologic homology with humans, including brain, digestive tract, musculature, vasculature, and an innate immune system [1–6]. Moreover, approximately 70 % of all human disease genes have functional homologs with the species [7]. Zebrafish are prolific reproducers with the potential to produce over 100 embryos per clutch. Their extrauterine development is rapid; the major organs of the zebrafish are fully developed by 24 hours post fertilization (HPF), and they are ready for use in larvae experiments by 3 days post fertilization (DPF). Zebrafish larvae are transparent during the early stages of life (through to 7 DPF), and this phase can be extended to 9–14 DPF by the addition of melanin synthesis inhibitor [8]. Zebrafish are small in size and require inexpensive food. It is easy, therefore, to maintain thousands of larvae in a laboratory at a reasonable cost.

Due to the advantages of genetic homology, physiology, and developmental similarity, zebrafish have increasingly become a desirable tool for studying the development and modeling of human disease [9, 10]. In the transparent embryo and larvae, clear time-lapse non-invasive imaging and protein/cell marker tracking significantly aid the observation of biological and disease processes [11, 12]. Several types of gastrointestinal disorders, such as inflammatory bowel disease, non-alcoholic fatty liver disease (NAFLD), and alcoholic liver disease, can be modeled in zebrafish [13–17]. Zebrafish have also been used in the analysis of complex brain disorders and muscle disease, including depression [18], autism [19], psychoses [20], and muscular dystrophies [21]. In addition, the ability to regenerate both fins and cardiac tissue make zebrafish particularly suitable for studying the wound healing response to various injuries [22].

Because of these advantages, zebrafish have proved to be superior for use in cancer research over the last decade. There are several long-standing methods for establishing a cancer model in zebrafish, including carcinogenic treatment, transgenic regulation, and the transplantation of mammalian tumor cells [23]. By inducing different gene mutations or activating signaling pathways through the use of chemicals, tumors can be induced in a wide variety of organs in zebrafish, such as the liver, pancreas, intestinal canal, skin, muscle, vasculature, and testis [24–28]. Transgenic technology enables the formation of specific types of tumor by the overexpression of particular oncogenes. The xenotransplantation of mammalian tumor cells into zebrafish provides a novel way of studying the interactions between the transplanted tumor cells and the host’s vasculature. Zebrafish have also been exploited for the investigation of tumor angiogenesis, which represents a critical step in tumor progression and is a target for anti-tumor therapies. The vascular system in a zebrafish embryo bears a strong resemblance to that in humans, and rapidly forms a single blood circulatory loop at 24 HPF. In zebrafish, the vascular endothelial cells can be stained by a fluorescent protein so that the neovascularization in the tumor microenvironment can be observed in the earliest stage. Tumor metastasis has also been modeled in zebrafish. The fluorescent-stained tumor cells are highlighted in the transparent zebrafish embryos and larvaes, so that the process of metastasizing tumor cells can be accurately tracked at the cellular level. The novel *casper* zebrafish line, a generation of double pigmentation mutant, even has a completely transparent body in adulthood. This superior generation of zebrafish, in conjunction with fluorescent imaging techniques, allows the noninvasive tracing of stained tumor cells in adult fishes [8]. It is worth mentioning that cancer stem cells account for only a small fraction of tumor cells and are too few in number to be feasibly transplanted in a mammalian model in order to assess metastasis. However, only a very small number of cancer stem cells are required in zebrafish for this purpose because of their small size. Additionally, the high fertility and low maintenance costs of zebrafish makes them suitable for the large-scale screen of antineoplastic drug efficacy and toxicity.

This paper focuses attention on the wide application of zebrafish as a superior model in cancer research, particularly with regard to establishing tumor models, and studying angiogenesis, metastasis, and antineoplastic drug screens.

### Cancer model establishment in zebrafish

Neoplasia was rarely found in wild zebrafish. Using a combination of chemical treatment, genetic technology, and tumor cell xenotransplantation, the vast majority of human tumors can be modeled in zebrafish [29]. Carcinogenic chemical treatment is commonly used in inducing tumorigenesis. Several carcinogenic compounds are able to induce canceration in a number of organs, such as dimethylbenzanthracene (DMBA) [30], diethylnitrosamine (DEN) [23], N-nitrosodimethylamine (NDMA) [31], N-ethyl-N-nitrosourea (ENU) [24], and N-methyl-N^1^-nitro-N-nitrosoguanidine (MNNG) [32]. The induced tumors cover a wide spectrum of tumors found not only in the digestive system (i.e. liver, pancreas, and intestinal canal) but also in the skin, muscle, vasculature, and testis [24–28]. As reported, exposure of the *vhl*^+/−^ zebrafish to DMBA revealed an increase in the occurrence of hepatic, bile duct, and intestinal tumorigenesis at 2 months following treatment [33]. Exposure to DEN results in different types of hepatocellular carcinomas, hepatoblastomas, hepatoma, cholangiocarcinoma, and pancreatic carcinoma in zebrafish [26]. Exposure of zebrafish to NDMA for 2 months leads to cholangiolar tumors (cholangiocarcinomas and cholangiomas) and hepatocellular tumors (hepatocellular carcinomas and adenomas) [31]. And exposure of zebrafish to ENU and MNNG results in liver and testis tumorigenesis [24, 27].

A number of reverse genetic tools have been developed for the study of gene functions in zebrafish. Morpholinos are usually injected at the 1–4 cell stage of embryos to provide transient knockdown of the target gene expression [34]. Another targeted genome modification technology, called TILLING (Targeting Induced Local Lesions IN Genomes), is highly dependent on large-scale traditional post-transcriptional forward genetic screens expression [35–38]. Moreover, engineered endonucleases, including ZFNs (zinc finger nucleases), the CRISPR-Cas system, and TALENs (transcription activator-like effector nucleases), provide efficient strategies to disrupt site-directed genes by inducing double strand breaks in the target genes [39, 40].

Several types of tumor have been generated by inducing mutants in known tumor suppressor genes. The knockout *p53* gene in zebrafish, for example, was found to result in an increase of malignant peripheral nerve sheath tumors (MPNST) [41]. In addition, the *APC* gene mutant in zebrafish leads to colon adenoma initiation and progression, suggesting an association with the activation of the Wnt signaling pathway [42]. Several other gene mutants were found to be related to different types of tumors in zebrafish. As reported, mutants in the *NF1* gene lead to high-grade gliomas and MPNSTs [43], those in *BRCA2*, *mybl2*, and *espl1* lead to testicular neoplasias [44, 45], those in the *pen*/*lgl2*, *bmyb* and *cds* genes cause epidermal neoplasia [32, 46, 47], and *GSTT1* deletion related to lymphoma progression [48], and *vhl* mutants lead to an increase in hepatic and intestinal tumors [33]. The immune and hematopoietic system in zebrafish is similar to that in humans, which means that not only solid tumors but also hematologic malignancies can be modeled [6]. The most frequent mutant in the tumor suppressor *pten* in zebrafish was related to an increasing morbidity of T-cell acute lymphoblastic leukemia (T-ALL) and hemangiosarcoma [49, 50].

Through the transgenic expression of human or mouse oncogenes, several cancer models have been established in zebrafish. T cell acute lymphoblastic leukemia was the first cancer induced by transgenic technology in zebrafish, which was induced by the Myc transgenes [51]. Subsequently, overexpression of the oncogenes *xmrk*, *Myc* and *KRAS*^(*V12*)^in zebrafish was found lead to hepatoma formation in both juvenile and adult transgenic fish [52–55]. Amplification of *MYCN* and *fgf8* expressions markedly promotes the formation of neuroblastoma [56]. Rhabdomyosarcoma has also been induced in zebrafish by using a specific up-regulate oncogenic *KRAS*^(G12D)^ expression [57]. Overexpression of *Akt1* enhances lipoma formation [58]. In combination with the *p53* mutant, overexpression of some oncogenes in zebrafish leads to different tumor phenotypes, such as *scr* (hepatoma) [59], *NRAS* (melanoma) [60], *BRAF* (melanoma) [41], and *EWS*-*FIL1* (Ewing's sarcoma) [61]. Additionally, the co-activation of the hedgehog and AKT pathways promotes tumorigenesis, suggesting that a transgenic approach is a useful tool for studying the interaction of oncogenes and oncogenic pathways in zebrafish [62].

Xenotransplantation represents a novel method to establish tumor models in zebrafish. One of the great strengths of xenotransplantation is that the transplanted tumor cells can be marked by fluorescent staining to enable them to be distinguished from normal cells in order to allow clear observation of the development process of the tumor [63]. The first human xenotransplant assays in zebrafish began in 2005. By injecting 1 ~ 100 melanoma cells into 3.5 ~ 4.5 HPF embryos, the migration in the developing larvae was clearly observed [64]. Transplantation of different types of tumor cells in zebrafish was carried out subsequent to this innovative work. Microinjecting glioma stem cells into the embryonic yolk sac region in 2 DPF embryos resulted in an observable invasion in the embryos via the vessels [65]. Hepatocellular carcinoma (HCC) was also modeled for the identification of the curative effect of anti-cancer molecules [66]. Several other types of tumor, such as lung cancer [67], pancreatic cancer [68], ovarian carcinomas [69], breast cancer [70], prostate cancer [71], retinoblastoma [72], and leukemia [73], have also been transplanted in zebrafish.

All the methods and types of induced tumor are combined in Table 1. The induced tumors are mainly located in the digestive and reproductive systems, and then the nervous system and epithelium.

**Table 1:** Summary of the methods used and the types of tumor induced in zebrafish

Technology	Treatment	Types of induced tumor	Reference
Chemical treatment	DMBA	hepatoma, cholangiocarcinoma and intestinal cancer	[30]
	DEN	hepatoma, cholangiocarcinoma and pancreatic carcinoma	[23]
	NDMA	hepatoma and cholangiocarcinoma	[31]
	ENU	hepatoma and testicular cancer	[24]
	MNNG	hepatoma and testicular cancer	[32]
Genetic technology			
Knockout:	*P53*	malignant peripheral nerve sheath tumors	[41]
	*APC*	colon adenoma	[42]
	*NF1*	gliomas and malignant peripheral nerve sheath tumors	[43]
	*BRCA2*, *MYBL2*, *esp11*	testicular cancer	[44, 45]
	*pen*/*lgl2*, *bmyb and cds gene*	epidermal cancer	[32, 46, 47]
	*GSTT1*	lymphoma	[48]
	*vhl*	hepatoma and intestinal cancer	[33]
	*pten*	T-cell acute lymphoblastic leukemia and hemangiosarcoma	[49, 50]
Overexpression:	*Myc*	T-cell leukemia and hepatoma	[51, 53]
	*xmrk* and *KRAS* ^(*V12*)^	hepatoma	[52, 54, 55]
	*MYCN* and *fgf8*	neuroblastoma	[56]
	*KRAS* ^(G12D)^	rhabdomyosarcoma	[57]
	Akt1	lipoma	[58]
	*Scr in p53 mutant background*	hepatoma	[59]
	*NRAS*, *BRAF in p53 mutant background*	melanoma	[60, 41]
	*EWS*-*FIL1 in p53 mutant background*	Ewing's sarcoma	[61]
Xenotransplantation	Transplant tumor cells in zebrafish	Melanoma, glioma, hepatoma, lung cancer, pancreatic cancer, ovarian carcinomas, breast cancer, prostate cancer, retinoblastoma, leukemia	[64–73]

### Tumor angiogenesis in zebrafish

Angiogenesis is considered a key factor in tumor growth and subsequent metastasis. Tumor vessels play an important role in transporting oxygen and nutrients to support the growth of tumor cells. For this reason, the capability of blood vessel formation within the tumor not only determines the malignancy of the cancer but also influences the therapeutic effects and prognosis. Both in research evidence and clinically, angiogenesis inhibitors in combination with chemotherapy improved the outcomes in cancer patients [74]. However, it is difficult to detect the original vascularization in traditional mammalian models because such models only permit the capture of static images, which probably relate to the late stage of the tumor. The lack of observation at the earliest stages of tumor formation means that the mechanism of vascularization is still not fully understood.

Human umbilical vein endothelial cells (HUVEC) are widely used in the investigation of angiogenic mechanisms in vitro. The system of angiogenesis can be evaluated by the cellular responses of HUVEC, such as cell proliferation, cell cycle, tube formation, cell migration, and cell adhesion to matrix proteins [75]. Several other quantitative angiogenesis assays, for instance the matrix implant assay and microcirculatory preparations such as the chicken chorioallantoic membrane and corneal micropocket assay, provide continuous monitoring of the angiogenic response [76]. However, the physiological status of angiogenesis may be quite different when translated to the area of cancer research. Indeed, angiogenesis in the tumor microenvironment relys on a distinct signaling pathway and displays large alterations in morphology and function when compared with normal vasculogenesis. Thus, in vitro research may be not suitable for modeling angiogenesis in tumor organization.

Zebrafish provide an ideal in vivo model for the research of tumor angiogenesis. The physiology and pathology of tumor angiogenesis in zebrafish is similar to that in humans because the tumor microenvironment in zebrafish is strikingly similar [77]. Additionally, the zebrafish vasculature grows rapidly (a single blood circulatory loop in zebrafish is fully developed in 24 HPF) and the transparent body allows for high-resolution in vivo non-invasive imaging [77]. The addition of PTU (a tyrosinae inhibitor that prevents melanin synthesis) to water can lengthen the transparency of the larvae to 9–14 DPF [78].This years, a pigmentation mutant *casper* line with a completely transparent body has allowed the non-invasive imaging of the vasculature across the whole body [8]. Real-time observation of vessels in larvae can be achieved after microinjection of chemical dyes into the vascular system [79]. Additionally, taking advantage of the Tg(flk1: EGFP) zebrafish, a transgenic fish line with a green fluorescent protein tissue-specific expression in the vasculature, individual cell growth and vessel formation can be easily detected under confocal microscopy [80]. In red fluorescent tumor tissues, the green fluorescent protein marked neovascularization is highlighted and enables the observation of angiogenesis in the initial stages.

Gene identification plays a key role in the exploration of angiogenesis and in discovering novel therapeutic targets for anti-angiogenesis drugs. Zebrafish are compliant to genetic manipulation at low cost and within a short time. In this manner, a number of signaling pathways for angiogenesis and various targets for drug treatment have been identified over the past few years. Targeted gene knockdown of TNFRSF1B in zebrafish was found to promote the apoptotic program, and knockdown of TNFRSF1A, or up-regulation of NF-κB, prevented endothelial cell apoptosis, suggesting that TNFRSF1A and TNFRSF1B were involved in the signaling pathways of angiogenesis [81]. In another study, a silencing of *LIM* kinases in pancreatic cancer tissues resulted in a decrease of angiogenesis in zebrafish [82]. These data suggest new therapeutic targets for the control of the tumor-driven angiogenesis process.

Compared with other angiogenesis models such as the chorioallantoic membrane of the chicken embryo, zebrafish show their superiority with regard to modeling the in vivo environment, compliance in genetic manipulation, and allowing clear observation of the interaction between tumor cells and neonatal micrangium.

### Tumor metastasis in zebrafish

The large amount of evidence from various studies has clarified that metastasis is a dynamic, complex, and multi-step process that includes tumor cells penetrating into the circulatory system, spreading to distant tissues, engrafting in the parenchyma, and developing in the graft area [83]. An insight into the mechanism of tumor metastasis is conducive to the discovery of anti-tumor drugs and the improvement of clinical treatments. Much of the previous analysis of metastasis conducted in in vitro cell systems had obvious weaknesses because the complete metastasis process cannot be abstracted away from the in vivo environment and vascular system. In vivo mouse models also have significant disadvantages: 1) it is difficult to evaluate the early stage of metastasis; 2) the complete process of metastasis in a mouse requires a long period of time; 3) real-time imaging of minute tumor lesions in deep tissues is impossible without termination and autopsy; 4) immunodeficiency mice may still have a residual anti-tumor competence that can prevent tumor cell metastasis [84]; 5) mice require feeding at high cost throughout the experiment.

The cancer model of zebrafish overcomes the drawbacks of murine xenograft models and shows several exceptional strengths. The adaptive immune system in zebrafish larvae is not completely developed until 14 DPF so that most transplanted cancer cells can survive and metastasize [85]. The transparent body of zebrafish enables the clear observation of tumor metastasis under the microscope. In the transparent *casper* line, the dynamic and spatial characteristics of micrometastases can be real-time imaged at the single cell level [8]. In order to highlight metastasis in zebrafish, tumor cells can be stained by a chemical dyestuff (such as CM-Dil) or labeled by red fluorescent protein (RFP) [86]. By injecting red fluorescent mammalian tumor cells into the Tg(*fli1*: EGFP) transgenic zebrafish, in which vascular endothelial cells are labeled by green fluorescent protein, both the process of tumor cell metastasis and changes in the vascular system can be clearly seen throughout the body. In addition, cancer stem cells are too few in number to be transplanted in mammalian models but zebrafish are small enough for such xenografting, and the rapid progress of metastasis in zebrafish is able to be observed within 2 days after injection [65].

Zebrafish provide an experimentally tractable animal model for the identification of suppressing or promoting factors in metastasis. By transplanting RFP expressing U87 glioma stem cells (GSCs) into the yolk sac of Tg(fli1:EGFP)^y1^ zebrafish embryos, the different invasive stages of GSCs, such as approaching, clustering, invading, migrating, and transmigrating, can be clearly observed at 2 days post-injection [65]. In this experiment, invasive GSCs were found to have MMP-9 high expression in common and treatment with the MMP-9 inhibitor significantly decreased the percentage of invasive cells in the embryos [65]. In an experiment on hypoxia, DiI-labeled tumor cells were injected into the perivitelline space of 48 HPF embryos, which were subsequently placed in hypoxic water for 3 days. A significant increase in metastasis and angiogenesis was detected using a fluorescent microscope at the single-cell level [87, 88]. Tumor cells and immune cells have been co-implanted in the same zebrafish to investigate the interactions in the tumor microenvironment [89]. The co-implanting of DiI-labeled tumor cells and DiD-labeled tumor-associated macrophages (TAM) relates to an increase in metastasis in zebrafish, and their association could be detected in overlapping colors [89].

The signaling pathway for metastasis has been evaluated in the zebrafish model. The technology of target knockdown of proteins involved in the signaling pathway with chemical inhibition or small interfering RNA is not new in zebrafish. The TGF-beta signaling pathway was found to control human breast cancer metastasis in zebrafish. After treatment with the TGF-beta signaling pathway inhibitor, the invasion and metastasis processes in zebrafish were inhibited significantly [70, 90].

### Drug screening in zebrafish

The effects of molecule antineoplastic drugs have often been detected by biochemical assays or in cell line models, but the outcomes were unsatisfactory. Because of a lack of a complete biologic context in the screening process, the identified active compounds were often ineffective when applied in a vertebrate model. At this point, a whole animal screen sheds valuable information on anti-tumor effects, organ toxicity, and pharmacokinetic data based on the entire organism [91]. However, mice are fiscally prohibitive for large-scale screen. Zebrafish, on the other hand, have emerged as a powerful platform for use in high-throughput antineoplastic drug screening on the strength of the following advantages. A pair of zebrafish produce hundreds of embryos a week, and larvae have a small size that can be arrayed in a 96-well plate, which greatly decreases the cost of maintaining them in the laboratory. Drug treatments can be easily achieved by merely adding the medicine to the aqueous environment. In addition, the transparent zebrafish body enables the real-time non-invasive imaging of anti-tumor effects and drug toxicity.

Most types of cancer can be modeled in zebrafish, thus zebrafish can be used to assess the anti-tumor effects of chemotherapeutic drugs. The growth of tumor cells and degree of invasion are the main concerned outcomes. Over the past 5 years, several large chemical screens have been performed in zebrafish. The anti-melanoma chemical genetic screen is one of the best representations. To our knowledge, the propagation of melanoma is critically related to the neural crest lineage. 2,000 chemicals were screened to identify inhibitors of the neural crest lineage in zebrafish embryos, and the selected chemicals were tested for effects in melanoma. Leflunomide, an inhibitor of dihydroorotate dehydrogenase, was found to inhibit the development of both neural crest and human melanoma. This screen shed light on the important role of zebrafish in antineoplastic drug discovery [92].

An anti-leukemia compound screen was performed in zebrafish in 2012. Zebrafish show a striking similarity in the hematopoietic system development with humans, and almost all human adult blood lineages have corresponding homologous cell lines in zebrafish. For this reason, effective hematopoietic drugs in zebrafish may serve the same function in humans. More than 25,000 small compounds were identified in this drug screen and, finally, a compound called lenaldekar (LDK) was found to be able to induce long-term remission in adult zebrafish with T-cell acute lymphoblastic leukemia (T-ALL). A subsequent study showed that LDK had a generalized anti-leukemia effect not only to T-ALL but also to several diverse leukemias such as B-ALL and CML [93, 94].

Anti-angiogenesis drugs have been screened in zebrafish. Following a screen of 288 new compounds, two kinase inhibitor compounds were found to have anti-angiogenic properties and a phosphorylase kinase subunit G1 (PhKG1) was identified as the kinase target [95]. In a similar way, rosuvastatin was identified as inhibiting the angiogenesis in developing zebrafish embryos [96]. Anti-lymphatic drug compounds were also identified in zebrafish. Four compounds previously used in humans were found to have anti-lymphatic activity in zebrafish [97]. These studies demonstrate that zebrafish provide an effective utility platform for large-scale antineoplastic drug screens and medicine efficacy detection.

Zebrafish have been used to identify compounds that work in the genetic signaling pathways of carcinogenesis. The *bmyb* gene is important for controlling the mitotic checkpoint and is connected with cancer susceptibility [32]. In order to identify the drug function of small molecules in the *bmyb* pathway, 16,000 compounds were tested. A compound named persynthamide was noted to have an inhibiting effect in *bmyb*-dependent mitotic defects and reduced the incidence of tumors in zebrafish [39].

Antineoplastic drug toxicity can be observed over a short period because of the rapid development of zebrafish. In a screen for detecting the inner ear hair cell toxicity of anti-tumor drugs, 13 out of 88 anti-tumor drugs, and 5 out of 10 drug combinations, were authenticated ototoxic. In addition, dose–response studies were performed on these detected drugs [98]. Several outcomes were usually detected to assess the toxic effect of antineoplastic drugs, such as cell damage, development process, and vitality. Drug toxicity screening has an important significance in selecting the appropriate therapy in clinical cancer treatments.

## Discussion

**Fig. 1:**
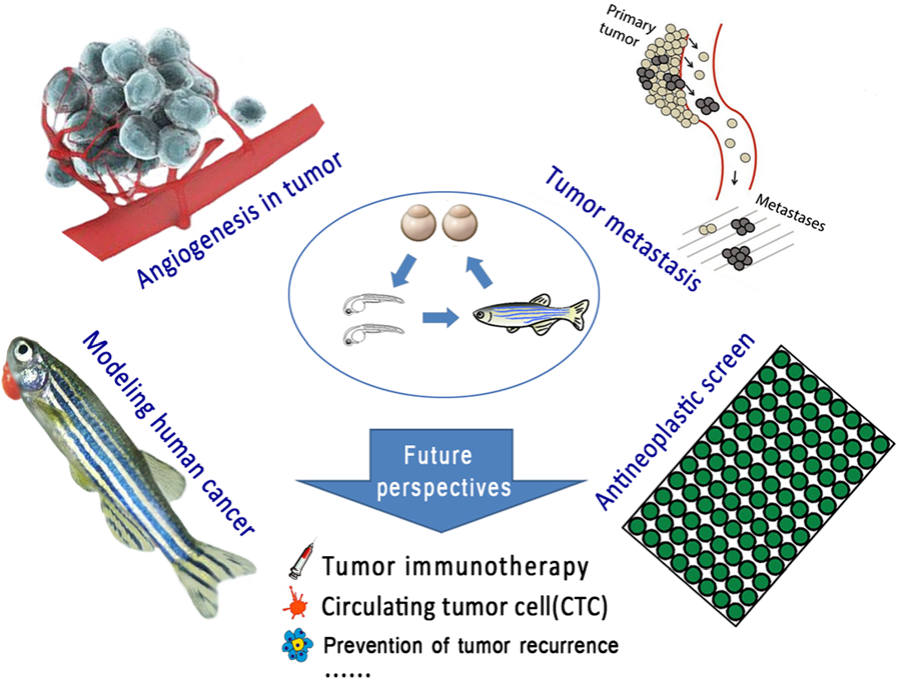
The main fields of application of zebrafish in cancer research

Considering the past two decades, it is easy to see that enormous progress has taken place with regard to zebrafish in terms of modeling human cancers. The wide use of zebrafish sheds greater light on the investigation of gene functions, tumor angiogenesis, tumor metastasis, and the discovery of antineoplastic drugs in cancer research (Fig. 1).

Several problems often trouble cancer researchers. Traditionally, the murine system is the most utilized animal system for studying human cancers. However, the long gestation time greatly lengthens the experimental procedure. In addition, real-time imaging of minute tumor lesions in deep tissues is difficult in a murine system unless autopsy is performed. The high cost of a murine system also rejects the high-throughput screening of drug discoveries.

There is no doubt that zebrafish will play an increasingly significant role in cancer research. Zebrafish have several prominent advantages in modeling human cancers, such as rapid development, a transparent body, high genetic homology, and ease of genetic manipulation. These unique advantages enable primary studies to be assumed before further verification using costly murine systems. Indeed, the ease of establishing a cancer model and the real-time observation of tumor progression in zebrafish greatly improves the efficiency of the experiment. Zebrafish provide a good model for researching the mechanisms of tumor angiogenesis and metastasis because the newly-formed vessels and metastatic tumor cells can be clearly marked and real-time observed through the transparent body. Additionally, the high reproductive rate and low financial cost of zebrafish enables high-throughput screening of anti-tumor drugs.

However, as an underdeveloped model organism, several weaknesses limit the application of zebrafish in the research community. Only a few versatile tools and validated reagents are suitable for use in zebrafish, in comparison with traditional mammalian models. This prevents us from exploring the details of the molecular and cellular mechanisms involved in cancer development. Zebrafish also have many duplicate genes that significantly complicate genetic manipulation. Forward genetic manipulation is not able to completely knock out both copies of the target genes, and any gene copies remaining will render the phenotype unchanged. CRISPR, a newly developed reverse genetic technology, shows its superiority to overcome this difficulty, as it has the property to remove multiple genes thereby reveal phenotypes [99]. In addition, it is difficult to give some water-insoluble drugs to zebrafish because the carrier solvents may be toxic before the drugs dissolve. Moreover, the embryos need to be raised in water maintained at 28 °C, and this temperature may not be optimum for the metabolism of mammalian tumor cells (which require 37 °C). Therefore, raising the incubation temperature to 34 °C may offer an effective compromise. As previously reported, maintaining embryos with transplanted tumor cells in 34 °C water did not affect the vitality of the embryos or the growth of tumor cells [100].

This paper systematically expounds the significance of zebrafish within the field of cancer research. It is a comprehensive summary of the various uses of zebrafish within the field and forecasts a more extensive application of them in the future. Tumor immunotherapy represents a new prevailing therapy in the clinical treatment of tumors. Zebrafish share a similar immune system with humans, so there is, therefore, the prospect of them being used to research the curative effects of tumor immunotherapy. Several other areas, such as tumor recurrence and circulating tumor cells research, will also require a zebrafish model for further exploration of the mechanism and physiologic processes.

In conclusion, zebrafish are increasingly becoming a superior vertebrate model for cancer research and can be expected to provide further contributions to our deeper understanding of the mechanisms of genetic function, angiogenesis, metastasis, and antineoplastic drug screening in the near future.
